# Treatment-seeking and uptake of malaria prevention strategies among pregnant women and caregivers of children under-five years during COVID-19 pandemic in rural communities in South West Uganda: a qualitative study

**DOI:** 10.1186/s12889-022-12771-3

**Published:** 2022-02-21

**Authors:** Ivan Mugisha Taremwa, Scholastic Ashaba, Rose Kyarisiima, Carlrona Ayebazibwe, Ruth Ninsiima, Cristina Mattison

**Affiliations:** 1 Clarke International University, P.O Box 7782, Kampala, Uganda; 2 Mbarara University of Science and Technology, P.O Box 1410, Mbarara, Uganda; 3 Rwamanja Refugee Settlement, Kamwenge District, Uganda; 4 McMaster Midwifery Research Centre, Hamilton, Canada

**Keywords:** Malaria prevention, Pregnant women, Children under-five, COVID-19, Uganda

## Abstract

**Background:**

Despite efforts to avert the negative effects of malaria, there remain barriers to the uptake of prevention measures, and these have hindered its eradication. This study explored the factors that influence uptake of malaria prevention strategies among pregnant women and children under-five years and the impact of COVID-19 in a malaria endemic rural district in Uganda.

**Methods:**

This was a qualitative case study that used focus group discussions, in-depth interviews, and key informant interviews involving pregnant women, caregivers of children under-five years, traditional birth attendants, village health teams, local leaders, and healthcare providers to explore malaria prevention uptake among pregnant women and children under-five years. The interviews were audio-recorded, transcribed and data were analyzed using thematic content approach.

**Results:**

Seventy-two participants were enrolled in the Focus Group Discussions, 12 in the in-depth interviews, and 2 as key informants. Pregnant women and caregivers of children under-five years were able to recognize causes of malaria, transmission, and symptoms. All participants viewed malaria prevention as a high priority, and the use of insecticide-treated mosquito bed nets (ITNs) was upheld. Participants' own experiences indicated adverse effects of malaria to both pregnant women and children under-five. Home medication and the use of local herbs were a common practice. Some participants didn’t use any of the malaria prevention methods due to deliberate refusal, perceived negative effects of the ITNs, and family disparity. The Corona Virus Disease-2019 (COVID-19) control measures did not abate the risk of malaria infection but these were deleterious to healthcare access and the focus of malaria prevention.

**Conclusions:**

Although pregnant women and caregivers of children under-five years recognized symptoms of malaria infection, healthcare-seeking was not apt as some respondents used alternative approaches and delayed seeking formal healthcare. It is imperative to focus on the promotion of malaria prevention strategies and address drawbacks associated with misconceptions about these interventions, and promotion of health-seeking behaviors. As COVID-19 exacerbated the effect of malaria prevention uptake and healthcare seeking, it’s critical to recommit and integrate COVID-19 prevention measures in normative living and restrict future barriers to healthcare access.

**Supplementary Information:**

The online version contains supplementary material available at 10.1186/s12889-022-12771-3.

## Background

Infection with malaria possess a major public health challenge due to the associated morbidity, high cost of expenditure, and increased risk of mortality [1]. Its burden was reported at 229 million cases worldwide in 2019; of which sub-Saharan Africa accounted for over 90% of the cases [2]. Moreover, 11.6 million pregnant women in sub-Saharan Africa were infected with malaria [2]. In Uganda, malaria remains a perennial infection affecting an estimated 95% of the population [3], and it remains the primary cause of 15–20% of inpatient treatment, 30–50% outpatients’ care and up to 20% of all mortality [4].

Whereas global concerted efforts to control malaria have been intensified in the previous two-decades, limited success has been realized as its epidemiological burden has surpassed past incidences [2]. More, the United Nations Sustainable Development Goals (SDGs) report that focused on goal-3 asserts that interruption in service delivery due to high illnesses and deaths from communicable diseases may warrant a 100% increase in malaria deaths in sub Saharan Africa [5]. Further evidence by the global data affirms that response to malaria prevention has leveled off, and the disease burden had escalated in endemic settings [2]. This has necessitated a renewed focus on malaria prevention progress and end its catalyzed ‘high burden to high impact’ [2, 6]. As a result, strategies to avert a worrying risk of malaria and portend global efforts towards attaining SDG-3 are a priority [5, 7]. In this regard, the World Health Organization (WHO) Global Technical Strategy 2016–2030 initiative adopted novel strategies to fast-track malaria prevention and reduce the disease burden by at least 90% by 2030, and eventual elimination [7]. The new strategies involve primary vector control measures such as the use of insecticide-treated bed nets (ITNs), and where appropriate, in-door residual spray (IRS) with children under-five years and pregnant women as the focus [2, 8, 9].

Concurrently, a pandemic: the new corona virus disease-2019 (COVID-19) outbreak was declared by the WHO in March 2020 as a global pandemic. The seriousness of the pandemic necessitated an immediate response to curtail its spread and avert the associated effects. In Uganda, a series of vulnerability reduction and containment approaches including cessation of international passenger flights, closure of inland portals of entries for passengers with the exception of cargo drivers, closure of all learning institutions and other high congregation points, freezing of public and private means of transport, outlawing of all mass gatherings such as places of worship, institution of overnight curfew, and eventually a nationwide total lockdown was declared on 24^th^ March 2020 [10]. Further, a re-organization in the healthcare service delivery was needed diverting resources form other programs to direct them to COVID-19 control with limited consideration of their impact across other healthcare needs and services [11]. Presently, there is evidence of significant disruptions to essential healthcare services as a result of COVID-19 pandemic [11–13]. This has added to the malaria management burden. Malaria and COVID-19 infections have similar symptomatic presentation such as breathing difficulties, fever, acute headache, tiredness among others. Additionally, the outbreak of COVID-19 and use of restrictive measures to avert its spread limited access to healthcare, and instilled fear of visiting health facilities due to contagion. In addition, malaria prevention programs were disrupted due to the paradigm shift.

Despite the proven efficacy of prevention programs, the burden of malaria remains unacceptably high due to challenges related to new pyrethroid-resistant mosquito strains, and laxity in uptake of prevention measures [9, 14–19]. A 2016 field survey in a rural district in Uganda [17], showed high malaria cases in the communities, despite the household possession of ITNs. As Uganda works towards malaria elimination, it is key to explore the specific local determinants that predict malaria prevention uptake. To the best of our knowledge, there is limited research that has explored the determinants of malaria prevention strategies among pregnant women and children under-five years during COVID-19 pandemic. This study explored the factors that are influencing the uptake of malaria prevention strategies among pregnant women and children under-five years in a rural district in south western Uganda during the COVID-19 pandemic.

## Methods

### Study design, site, and duration

This was a qualitative, explanatory single case study using focus group discussions, in-depth interviews and key informant interviews as the main sources of evidence. The case was defined as the common case conducted in Birere sub-county located in Isingiro district, southwestern Uganda between August to November 2020, to garner insights into the factors that influence the uptake of malaria prevention strategies among pregnant women and children under-five years [20]. Birere sub-county comprises 9 parishes and 76 villages, with a population of 26,000 people [2]. The study period overlapped with the second annual peak of the rainy season (September to November), and by this time, some of the instituted COVID-19 restrictions including in-country means of transport had been partially uplifted as of July 21^st^ 2020. Study activities were conducted in compliance with the COVID-19 guidelines.

### Study participants and sampling strategy

The study purposively enrolled participants aged 18 years or above who were pregnant and/or provided care for pregnant women and newborns and had lived in Birere sub-county in Isingiro district for at least 6 months. These included pregnant women, caregivers of children under-five years, community health workers (village health teams) and tradition birth attendants and local leaders. For example, although the antenatal care (ANC)-based healthcare providers (HCPs) offer ANC services including malaria prevention and treatment; traditional birth attendants (TBAs) remain pivotal in the communities, partly due to persistent gaps in rural HCP availability and continued preferences for home-based deliveries. The auxiliary nurse midwives (ANMs) provide primary healthcare in community-level clinics and they support maternal-child health care provision. The village health teams (VHTs) act as community liaisons for the promotion of primary health care services, while the local council (LC) leaders supported community mobilization. Informed by previous qualitative studies, in which saturation is typically reached after interviewing 6–12 individuals with similar backgrounds [18], this study conducted 8 focus group discussions (FGDs), 13 in-depth interviews (IDIs), and 2 key informant interviews (KIIs)0.3. The details of the respondents are summarized in Table 1.

**Table 1:** Showing the data collection methods and the different respondents

Data collection method	Number	Respondents
Focus Group Discussions	8	- 4 FGDs with caregivers of children under-five years - 4 FGDs with pregnant women
In-depth interviews	13	-2 IDIs with caregivers of children under-five years, - 2 with pregnant women, -2 with local leaders, -3 with health care providers, - 2 with VHTs, -1TBA -1 community social worker
Key Informant Interviews	2	-1 for the sub-county VHT coordinator -1 for the HC-III in charge

*Key:* *FGD* Focus Group Discussion, *IDI* In-depth Interview, *VHT* Village Health Team, *TBA* Traditional Birth Attendant, *HC* Health Centre

The study participants were recruited from the 8-parishes in which, four parishes were selected for FGDs among pregnant women, and an equal number were considered for caregivers of children under-five years. Another parish was considered for each KII and IDI. The parishes were selected randomly to eliminate bias. The one parish that was not considered in either FGD for pregnant women or caregivers of children under-five years was prioritized for the in-depth interview respondents. Assisted by the local council-1 leaders, the VHTs compiled a list of households with a pregnant woman or a child under-five years. The list was used to randomly select households that participated in the FGD. Each FGD was clustered at the parish, with each village represented. Participants to the IDIs and KIIs were identified and contacted by the VHT coordinator, and the study team then followed up with those who were willing to participate.

### Data instrument and collection

Guided by previous studies [21–25], data collection tools (supplementary files 1 and 2) were developed. FGD and KII questions focused on symptom recognition, healthcare seeking, knowledge, and behaviors towards malaria prevention. Also, the study assessed the impact of the COVID-19 pandemic on prevention uptake. The IDIs with pregnant women explored behavior to protect against malaria during pregnancy. On the other hand, IDIs with HCPs assessed the perceived behaviors of pregnant women and caregivers of children under-five towards malaria prevention uptake, and if malaria was emphasized during ANC. The interview guides were reviewed by two independent experts who were knowledgeable in the field of malaria. After the expert review, these were translated by 2-separate proficient persons who knew well both English and Runyankore-Rukiiga languages. Then, one of the research team members (CA) and the principal investigator (IMT) compared the translations, and compiled the final translated tools. These were then cross checked by two members of the team (RK and RN) for accuracy and comprehension in the Runyankore-Rukiiga language. The interview guides were pretested in communities within Mbarara City, Southwestern Uganda, and changes were made accordingly. Further, the interview guides were piloted in the first interview and edited during the data collection process in response to emerging themes. Additionally, participants’ socio-demographic information was captured.

Data collection was conducted by at least three members of the team supported by a research assistant who was conversant with the topic on malaria, and qualitative research methods. The research team liaised with the VHTs and LCs on the day of the appointment, and a convenient time as proposed by the participants was considered to convene. Each FGD was comprised of 8–10 participants in light of the COVID-19 guidelines. Individual introductions were done, and the research team sought individual written informed consent after explaining the purpose of the study. An interview guide in the local language (Runyankore-Rukiiga) was shared with the participants and guided the discussion with probing to pursue any emerging inquiry in major trends and cross-cutting themes. A member of the team led the discussion, and clarity to the question(s) was ensured by rephrasing where necessary. Participants were anonymized, and the discussion was guided by agreed rules to warrant appropriate communication. Field notes were recorded to contextualize the data and provide reflections on each interview, and the interviews were audio-recorded.

### Data management and analysis

The audio recordings were transcribed and translated into English if conducted in the local language (Runyankore-Rukiiga language). Transcripts were carefully and independently studied by two dedicated members of the team, and reviewed by the lead author to assess translation quality and fidelity. Transcripts were read and re-read to allow familiarization with the text, and brief notes were taken to document the emerging themes. A codebook was developed based on the original and emerging themes. Content analysis was used to conduct the initial data analysis, and NVivo 10 (QRS International) was used to guide data analysis based on the emerging themes and patterns. Data from varied participants and sources (FGDs, IDIs, KIIs, and observations) were extracted and triangulated by three members of the team. The emerging concepts were categorized based on the study objective, coded, and subjected to conventional content analysis using a thematic approach with typical and atypical statements identified for sub-themes to illustrate key findings.

### Ethical consideration

Ethical approval was obtained from the Mbarara University of Science and Technology Research and Ethics Committee (UG-REC-005) before the beginning of the study. Administrative permission was obtained from Isingiro district health office. The study obtained written informed consent, and permission to audio record the interviews from all participants. Legally authorized representatives (literate family member) of illiterate participants provided informed consent for the study. The anonymity of participants was ensured at all stages of data collection and analysis.

## Results

Eighty-six adult participants were recruited. Of these, 72 participants were enrolled in the FGDs, 12 in the IDIs, and 2 as key informants (KIs). Participants’ median age was 34 years (range: 18-57 years). About 98% percent of the participants (N = 84) were females and farming was the main source of livelihood (*N* = 61). Most participants were married (*N* = 66) with a median of 3 live-children, and the majority (*N* = 49) of the pregnant women were in their second gestation trimester. Participants had attained various levels of education as summarized in Table 2.

**Table 2:** Showing the demographic characteristics of the participants

Variable	Frequency	Percentage
Age category (Years)		
16–20	4	4.65
21–30	51	59.30
31–40	19	22.09
Above 41	12	13.95
Gender		
Female	84	97.67
Male	2	2.33
Marital status		
Single	5	5.81
Married	66	76.74
Separated	4	4.65
Divorced	7	8.14
Widowed	2	2.33
Cohabitation	2	2.33
Education level		
Primary	30	34.88
Secondary	18	20.93
Tertiary	15	17.44
No formal education	8	9.30
Religion		
Christian	61	70.93
Muslim	14	16.28
Traditional	2	2.33
Other (Seventh Day Adventist)	9	10.47
Employment status		
Employed	31	36.05
Self-employed	22	25.58
Unemployed	33	38.37
Occupation		
Farmer	41	47.67
Civil / Public servant	31	36.05
Trader/ businesswoman	14	16.28
Number of biological children		
1–3	29	33.72
4–6	38	44.19
Above 7	19	22.09
Gestation period		
First trimester	11	12.79
Second trimester	49	56.98
Third trimester	26	30.23

**Fig. 1:**
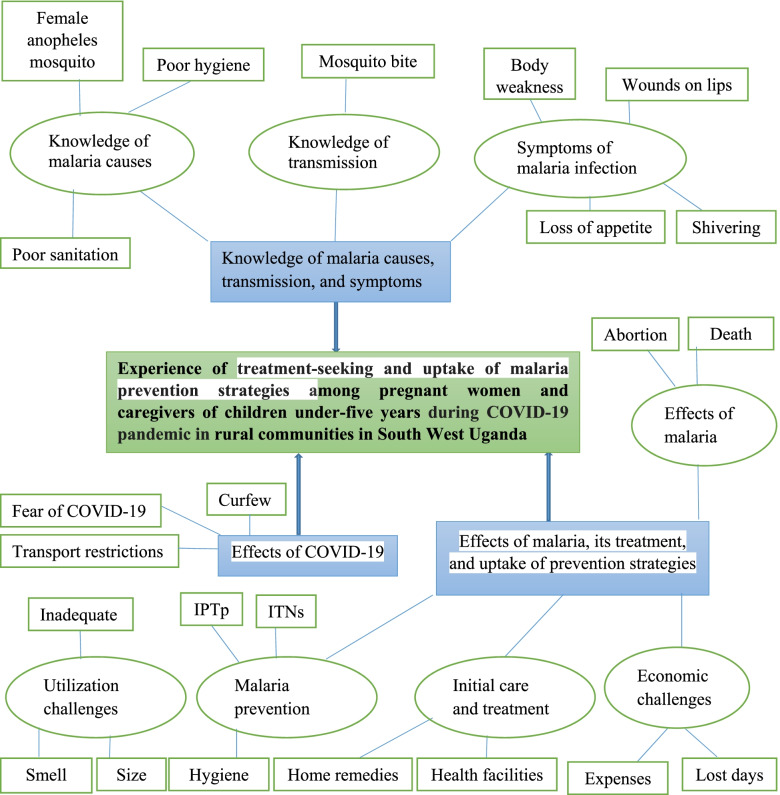
Showing illustration of the thematic analysis of the study. Key: ITNs: Insecticide Treated Nets, IPTp: Intermittent Presumptive Therapy during pregnancy

The key themes that emerged from the data were: (1) knowledge of malaria causes, transmission, and symptoms, (2) effect of COVID-19 pandemic on healthcare-seeking and the prevention of malaria behaviors, and (3) perceived effects of malaria, its treatment and uptake of prevention strategies. Each of these themes and subthemes are diagrammatically illustrated in Fig. 1, and are discussed in detail below.

### Knowledge of malaria causes, transmission, and symptoms

#### Knowledge of malaria, perceived cause and transmission of malaria

Malaria was perceived as the major health problem, and a common life-threatening disease among pregnant women and children under-five years. In their own experiences, participants indicated that infection in malaria was high during the rainy season, and the onset of the disease was sudden. In a participant’s view; *‘Malaria is a life-time disease, and is transmitted by an infected a mosquito bite while feeding on humans’. (26 years old, pregnant woman).*

Malaria was attributed to varied causes such as mosquito bites, poor sanitation, and hygiene, as well as harvest season of mangoes and maize.

Mosquito as a cause of malaria: Most study participants recognized that the female anopheles mosquito lived in bushy areas, heaps of rubbish, stagnant water, broken pots, water tanks, and open tins near homesteads, and is the causative agent of malaria as given in the narrative: *‘…when it rains, the female Anopheles mosquito begins to lay eggs, and the life cycle is completed when it feeds on human blood. Subsequently, as one fails to close the house entrances (doors and windows) early, mosquitos’ gains access to the house and unaware, they bite and infect a person at night if not protected. Biting occurs from one person to another; as a result, malaria is spread if one is infected’. (29 years old, pregnant woman).*

Poor sanitation and hygiene as a cause of malaria: Six of the participants from the different FGDs referred to malaria as a disease caused by ‘poor sanitation and hygiene’. One of the participants elaborated that; *‘…if one lives in a dirty environment, the bushy compounds, gardens near the homestead, garbage, and decomposing matter attracts many illnesses including malaria’. (24 years old, mother to 1 year old).*

The harvest season of mangoes and maize as a cause of malaria: Two participants from different FGDs narrated; *‘….since my childhood, I have always seen people suffer from malaria during the harvest time particularly for mangoes and maize’ (34 years old, mother of 2 children & 26 years old, pregnant woman).*

#### Symptoms of malaria infection

Participants locally recognized the symptoms of malaria infection as wounds on the lips, feeling cold, loss of appetite, yellowing of eyes, headache, general body weakness, shivering, and high body temperature. A pregnant woman described the symptoms of malaria infection as ‘a weird presentation’ and was associated with craving for good food as given in this narrative: *‘…malaria causes unease! You begin to shiver, vomit, and become weak similar to the early stages of pregnancy! This can stigmatize as it looks unusual in the public. Also, one develops a sour taste, and craves good foods! Sometimes you ask for rare foods like fish, and you are not yourself!’ (31 years old, pregnant woman)* The symptoms of malaria during pregnancy were described to resemble those of early pregnancy.

The caregivers of children under-five years considered the symptoms of malaria as ‘sudden changes to their wellbeing’ manifesting as diarrhea, unending crying, vomiting, and failure to feed as narrated; *‘…when a child suffers from malaria, s/he looks bad! S/he begins to vomit, cries endlessly, rejects all the foods and the as a mother you feel peaceless.’ (26 years old, mother to 3 years old)*. Further, the onset of malaria among children under-five was sudden, and affected their emotions and behavior. The symptoms were not obvious, and one relied on the change to predict the dire state of their health. A caregiver specified the difficulty to recognize these symptoms among younger children, as described: *‘…symptoms of malaria are challenging among children! From nowhere, a child starts to cry a lot, the body temperature rises suddenly, they develop diarrhea, and they pass yellow smelly urine. The child rejects foods, feels a burning sensation while breastfeeding, and often rejects the breast. They occasionally vomit yellow kinds of stuff’. (41 years old, mother of five)* More, malaria was associated with a change in behavior. There occurs a noticeably reduced activity such as playing among children, and a child would often fall sleep. The unending cry and high body temperatures associated with malaria among children under-five stirred swelling of the blood veins and led to the yellowing of eyes. These changes were considered to manifest later, and they would depict a late-stage of malaria infection which necessitated to seek for healthcare. Profoundly, parents who spent time away from their children didn’t recognize malaria symptoms in time, as reported; *‘…I had gone to work, and on my return, I found my 11-months baby very sick, and she had developed rigors, and if I had delayed to take her to the hospital, maybe the worst would have occurred’ (33 years old, pregnant woman).* These narratives showed a good recognition of malaria symptoms, however, to some, these required a caregivers’ critical attention.

### Effects of malaria, its treatment, and uptake of prevention strategies

#### Perceived effects of malaria infection

The participants stressed the effects of malaria as life-threatening to both pregnant women and children under-five years due to the risk of mortality, and its sudden onset that necessitates urgent attention as narrated by one of the participants: *‘…my child developed high temperatures. …I gave him a fraction of panadol, but the night was too long and worrying! Very early the next morning, I took him to a hospital where he was put on a drip (intravenous treatment) and he got well’. (28 years old, mother to 4 years old).*

On the sad note, a participant reportedly lost a child to severe malaria as narrated in the following quote: *‘…my child presented with signs of malaria, and in a short time, his condition worsened! He became very unconscious, and I anticipated many possibilities including death! Unfortunately, he didn’t make it even when we struggled to get to the hospital’. (38 years old, mother of four).*

Among pregnant women, participants were cognizant of the negative effects of malaria such as spontaneous abortions and stillbirths. Some pregnant women reportedly experienced peri-vaginal bleeding and resulted in pregnancy complications such as spontaneous abortion as a result of malaria. Resultantly, pregnant women greatly considered malaria prevention uptake, as narrated:

*‘…pregnant women do it to protect their unborn babies, not even for themselves! Because one knows that if she gets affected by malaria she could be treated and gets healed but what about the unborn child? if you lose it, you have lost it…’. (22 years old, pregnant woman).*

#### Economic challenges of malaria

Infection with malaria posed an economic challenge as it negated productivity. The reported effects were unfavorable as they negatively affected both the caregivers of children under-five years and pregnant women. Participants reported many days lost, and often led to huge expenditure on treatment bills, and this may lead to many debts.

#### Initial care and treatment for malaria

Most participants (*N* = 41) attested that usually when a child or pregnant woman becomes badly off, they used home-remedies and then rushed to the health facility for treatment. From there, they would get well or were referred to a higher health care facility. A participant narrated: *‘…I returned from the market and found my 2-year child unwell. Her elder sister whom I had left to baby-sit her informed me that she had vomited, and she had high temperatures. I then remembered that I had panadol in the house, and I gave her a small’ fraction of the tablet and I proceeded to the health center’. (35 years old, mother of three)* Also, a non-medicinal approach was used to subvert the high temperatures as narrated: *‘…I got a clean cloth and soaked it in cold water, after which, I massaged the child as I prepared to go to a clinic’. (23 years old, mother to 2 years old)* The use of home remedies was confirmed by the VHT who indicated that they are used to subdue the symptoms of malaria while still in the community before they seek healthcare. She elaborated that whenever a child or pregnant woman got sick, home remedies (tablets leftover from a previous dose, or one would buy them from a clinic) were used first. Also, it emerged that local herbs are used as narrated: *‘…there was when you find that you don’t have any tablet of panadol, and you don’t have any money with you! …so I went to the farm and searched for local herbs (‘eshabiko’), I boiled it and used it to massage, and gave some to drink. I then started looking for ways to go to the hospital when the child was somehow stable’. (37 years old, mother to 8 months old).* To explore further the use of herbal remedies, another participant narrated that: *‘…I went to the hospital, and they could not detect the disease my child was suffering from. I requested for referral, however, on my way, I felt like even where I will go, they won’t get the disease. So, I went to the elderly woman who gave me herbs for the baby to drink and she became better’. (31 years old, mother of three).*

#### Malaria prevention strategies

Participants knew and used varied malaria prevention approaches such as ITNs, insecticide home sprays, intermittent presumptive therapy during pregnancy (IPT) and emphasized homestead hygiene practices such as drainage of stagnant water, covering of water tanks, clearing of bushes around homesteads, destruction of broken pots and empty tins, as narrated: *‘…we practice varied malaria prevention methods, for example, most of us close our windows and doors early (by 7 pm), and ensure that our compounds are free from any water’. (36 years old, pregnant woman).*

As the most available and widely used preventive method, the availability and effectiveness of ITNs was rated high. Participants expressed positive attributes and indicated that distribution and sensitization efforts had yielded positively as narrated: *‘…. there is strong evidence that ITNS work for us. Even when you go for antenatal care visit, they emphasize malaria prevention, and they also give us an ITN. At immunization, young children are given a free ITN, and their use is welcomed by all of us’. (34 years old, pregnant women).* The ITNs were perceived to have reduced on the burden of malaria among the vulnerable groups, and majority of the participants expressed positive attributes towards their regular and correct use as narrated: *‘Ever since I started sleeping under an ITN, I have spent about four years without suffering from malaria. When people noticed that use of ITNs reduced malaria infection, they understood the importance of these nets, and loved using them’. (25 years old, pregnant woman).*

Despite this, ITNs were not 100% effective since it only offered protection while one is asleep. The methods of spraying with an insecticide were not common as narrated: *‘….sometimes you can’t afford to buy that mosquito spray, others fear using the spray because it causes severe headaches, and others are allergic, so you may use it and you start breathing badly or get a skin rash’ (27 years old, pregnant woman)*. Often, participants considered the use of ITNs as the only available method considering its (ITNs) availability and ease of use. Consequently, participants recognized the good attributes of ITN use, and they expressed an irresistible willingness to buy and use an ITN as narrated: *‘….ITNs were freely distributed by the government, and even if I was to buy one, it costs less than the treatment charge for malaria. While an ITN would cost about 10,000 Uganda Shillings (an equivalent of 2.78 United States Dollars), most of the private health facilities would charge an average fee of 68,000 Ugandan Shillings (an equivalent 18.89 United States Dollars); which is not affordable by the majority of the households, and it is more expensive if more than one individual in the household fell sick’ (43 years old, mother of six).* To this, HCPs and VHTs asserted the positive attributes of ITN use and affirmed the positive perception and improved practices among communities as narrated; *‘…when they would announce that they are distributing ITNs, people would leave all they were doing to make sure that they also get. During the distribution, they don’t mention bad things about them (ITNs) (38 years old, mother of five/midwife)*. The local leaders affirmed the positive attitude as given in the narrative: *‘…people have seriously taken up the call to malaria prevention. We usually monitor homes to find out whether the malaria prevention and control measures are practiced, and we are impressed by the majority’ (59 years old, father of eight/local council chairperson of P_cell).* Participants reported a positive attitude as given in the verbatim: *‘…malaria infection has reduced in our communities. Most of the people now suffer from cough and flu’ (47 years old, VHT)*. This was further affirmed by the healthcare provider who reported that: *‘…the cases of malaria have declined, and most of the patients we see at the health centre are due to respiratory tract infections’ (44 years old, mother of four/midwife).*

#### Utilization and challenges of malaria prevention methods

The use of ITNs was preferred as people freely acquired them from the universal rollout programs. Besides, ITNs are quite easy to use. The use of insecticide sprays was not common as the spray is costly for a rural population, and the chemical caused adverse effects if inhaled. Despite the varied strategies and positive attributes of malaria prevention, some participants didn’t utilize such measures. These were ascribed to deliberate refusal, being careless with their health, others felt suffocated under ITNs, uncomfortable on hot nights, while health conditions like asthma portended consistent use of ITNs. Other participants considered ITNs as highly flammable, which would put the house at risk, as narrated: *‘Some people are scared of using ITNs because they can easily catch fire and burn the whole house down, as it has been reported in most fire outbreaks in school dormitories’. (29 years old, mother of 3 months).* Also, family disparity as a wife may prefer sleeping under an ITN while the husband is uncomfortable was another reported factor for ITN non-use. More, some participants didn’t receive sufficient ITNs for all the members in their households as explained by the local council leader: *‘…usually, we distribute one ITN to two people in a household, so in the end, households that have many members sleeping in separate beds will have a problem of inadequate ITNs’. (40 years old, father of six/local council leader n_cell).*

### Effect of COVID-19 pandemic on healthcare-seeking and prevention methods

#### Effects of COVID-19 towards healthcare access for malaria infection

There were unprecedented challenges that affected healthcare access as narrated: *‘…at the start of the lockdown, public transport means were stopped and the curfew was put in place. These halted people accessing healthcare facilities’. (38 years old, mother of six)*. Further, two participants expressed dismay as reported: *‘… that even when the lockdown was eased, the private cars were allowed to carry a limited capacity, and even then, it was expensive to hire someone’s car to access the healthcare facility’. (28 years old, pregnant woman) ‘…in the subsequent easing of the transport restrictions, public transport was allowed, but to carry half the capacity of the passengers. […] this led to exorbitant transport charges’ (21 years old, mother to 2 years old).* To the greatest extent, COVID-19 negatively affected healthcare access. This was affirmed by local council authority as narrated: *‘…. when COVID-19 broke out, the world changed! The president stopped the movement of both motorcycles and cars. Whenever a child or even a pregnant woman fell sick, it was difficult to go to the hospital. Sometimes the motorcyclists would risk transporting you, but they would be badly beaten by the law enforcers. Also, the motorcyclists would charge much more money, and this discouraged people from accessing healthcare facilities’. (33 years old, father of three/local council leader q_cell).*

#### Effect of COVID-19 pandemic on malaria prevention methods

As the most available and commonly utilized malaria prevention method, the use of ITNs was negatively affected by the COVID-19 pandemic. For example, COVID-19 increased family conflicts, and these affected the availability and use of malaria prevention as narrated; *‘…the outbreak of Corona (COVID-19) increased family conflicts! When such happened, it made it hard to share a bed with ITN! So a woman and her baby would go and sleep somewhere else! As we received limited ITNs, when you shift from the main bed, then you sleep on a mattress that is put down on the floor without an ITN’ (29 years old, mother to 11 months old).*

Also, the sudden closure of schools with no clear understanding of when these would re-open for the learners posed great challenges in regard to ITN utilization. It emerged that most school supplies like ITNs were left behind, and as learners went back to their homes, resulting in a scarcity of ITNs there. For parents who did not want their children’s lives risked to mosquito bites, they gave in theirs to protect the children as narrated: *‘Due to COVID-19, schools closed abruptly and children left their ITNs at school. As a parent, it hurts to have an ITN when your three children who share a bed are not protected. So, I gave mine to protect my children, but now it is six months and schools haven’t opened. I think I will get another during my antenatal visit’. (31 years old, mother of three).*

Further, the COVID-19 pandemic shifted the focus of priorities as reported: *‘…some government programs including ITN distribution were halted, and people spent several months without ITNs. […] also, malaria-related training reduced due to COVID-19 as a gathering is discouraged’ (37 years old, mother of four/VHT).*

Furthermore, COVID-19 negatively affected household income, and this hampered malaria prevention as narrated: *‘…COVID-19 has reduced the utilization of malaria prevention using insecticide sprays as the standards of living were greatly impoverished as people lost jobs. …the major focus is on survival, and priorities have left households compromised with malaria prevention’ (23 years old, pregnant woman).*

On the contrary, the curfews put in place ensured early return to home (from 07:00PM to 06:00AM), and this allowed people to be indoors much earlier than before, and this reduced the risk of mosquito bites as given in the verbatim: *‘…. everyone now returns so early because of the curfew, so we ensure that we cook in time and go to bed. This protects us from mosquitoes that bites us late in the night’. (36 years old, pregnant women).*

## Discussion

The results of this study affirmed that the majority of participants knew that the causes and transmission of malaria were associated with mosquito bites, and further asserted that malaria was transmitted from person-to-person through a mosquito bite. This finding agrees with previous studies [26–28] and is indicative of ample community knowledge on malaria, which is key to its prevention. The observed malaria-related knowledge is ascribed to the awareness campaigns, and this is hoped to foster prevention uptake [29]. On the other hand, some participants did not correctly understand the causes, transmission, and symptoms of malaria which may impede prevention uptake, and risk community malaria infections. Also, the early signs of pregnancy overlapping symptoms of malaria have been reported [30, 31], and may negatively affect healthcare seeking [32–34].

Misconceptions about malaria prevention still exist and may portend malaria prevention uptake. For example, studies conducted in Nigeria reported that misconceptions related to malaria prevention methods affected their uptake [35, 36]. Mathania et al.[37] further assert that uptake of malaria prevention requires strategies that address misinformation on the various methods. Similar findings from Zimbabwe and Burkina Faso have affirmed this [36, 38]. Misconceptions related to malaria causation have indicated spurious prevention strategies, which risks the lives of the vulnerable populations [39, 40]. To this, overlapping knowledge on malaria causes, symptoms, and prevention has been observed between pregnant women and the caregivers of children under-five [41]. In Uganda, numerous studies have shown a knowledge gap in both malaria causes and prevention measures [17, 40]. In our experience of exploring the knowledge, attitude, and behavioral practices towards the use of ITNs among pregnant women and children under-five years in Isingiro district, there was optimal knowledge of malaria prevention, however, major barriers existed related to texture, color, and chemical composition impeded their utilization [17]. The low uptake of malaria prevention among vulnerable groups raises critical health concerns.

Participants were cognizant of the negative effects of malaria, and these are key towards malaria prevention uptake [23, 42]. Further, the reported effects were detrimental as they negatively affected both the caregivers of children under-five years and pregnant women [27, 31]. These corroborate with previous reports [43, 44], and these demonstrate the threat posed by malaria infection. Consequently, this shows the need to urgently seek healthcare to avert the fatal consequences of the disease. Further, although the majority of the participants who reported fever had sought formal healthcare, some had initially considered home-remedies in form of paracetamol, herbs, and tepid sponging before seeking formal health care. The alternative remedies were agitated by frequent drug stock-outs in public health facilities and exorbitant charges in private health facilities. This pattern of healthcare-seeking affected delivery as some pregnant women reportedly opted to deliver from their villages due to long distances, and transport barriers. This affirms a previous report [45], and further highlights the gap in timely healthcare-seeking [46]. COVID-19 control requirements made seeking outside medications more difficulty of finding and cost of transport and the decrease in income. This finding contravenes recommendations that oblige timely healthcare seeking [47]. Similar to our study, previous research reported participants adopting a thriving private market and caregivers practiced over-the-counter remedies [48]. This suggests that home treatment may provide a more attractive option than formal healthcare [49, 50], since access to shop‐bought medicines can be easier, with less time spent traveling or waiting, longer opening hours, and customer‐orientated staff [50]. Additionally, as malaria infection occurs most during the rainy season when farming is at its peak, the use of home remedies may be preferred. From this study, paracetamol (also known as panadol) as an antipyretic monotherapy was reportedly used to ameliorate fever as an initial home remedy. This practice is inapt and is attributed to the negative influence of media advertisement of various antipyretics that acclaim for home treatment of fever with the addendum that ‘the doctor’ should be consulted if symptoms persist. Resultantly, healthcare seeking is delayed with the hope of getting better, a risky practice that aggravates unnecessary drug side effects, severe life-threatening complications, and preventable deaths [51, 52]. Home and local remedies, as per opinions by participants were as a result of healthcare challenges, similar to previous studies [53, 54].

Participants knew and reportedly utilized more than one malaria prevention method. Human vector control through the use of ITNs was the most available and participants expressed irresistible willingness to its use; consistent with a previous report [55]. Despite this, some participants did not use them due to perceived negative sentiments linked to the adverse risks of the insecticide, which corroborates well with previous reports [17, 56]. On the other hand, insecticide spray presented severe adverse challenges only exacerbated by COVID-19’s negative impact on income and decrease in educational reminders because group gatherings to discuss health topics were curtailed. These health-impacting outcomes have been recognized, and previous studies have emphasized the need to limit children, and women's exposure to in-door insecticide use [57, 58]. Again, some participants didn’t uptake malaria prevention measures, and this was ascribed to the consideration of ITNs as highly flammable, deliberate refusal, being careless with their health, others felt suffocated under ITNs, uncomforted night as it would be too hot, while conditions like allergy and asthma affected consistent use of ITNs. Also, family disparity as a wife may like sleeping under an ITN while the husband is uncomfortable, and conflicts in homes. Previous research has shown similar findings [17, 59, 60], and these affirm a previous report [61] that the malaria prevention uptake is not effectively used. It is plausible that when the government is going to distribute ITNs, a single ITN-to- a person in a home is ideal. Also, they government ought to consider tough punishments for people who misuse the ITNs.

The outbreak of the COVID-19 pandemic presented an unprecedented challenge towards malaria prevention uptake. For example, some malaria prevention interventions like ITN distribution and malaria-related training were halted because of discouraging gatherings. The observed pattern has been reported elsewhere [62, 63]. Educational gatherings are helpful in supporting behaviors to prevent and control malaria. Also, movement restrictions reduced the mobility of healthcare workers in-country, limited the capacity of staff, community outreach activities, and logistical supplies that were supporting malaria prevention [64]. Furthermore, COVID-19 reduced functional health care facilities as healthcare workers were repurposed to support the COVID-19 control response [65]. Even when public transport resumed, there remained significant barriers particularly exorbitant transport fees as a result of a reduced carriage capacity, and less income to pay which hindered malaria prevention uptake particularly access to artemisinin combination therapies [64]. Further, increased time together increased family conflicts which negatively affected the consolidated use of malaria prevention. The findings of this study furthers affirms to the social and health impacts concluded in a commentary drawn on sub-Saharan African health researchers’ accounts of their countries’ responses to control the spread of COVID-19 [66]. These findings further underline the worrying negative effects experienced by the majority of the vulnerable population in rural southwestern Uganda. The findings of this study ought to be interpreted in light of the following: 1) the study interviewed caregivers to children under-five as the proxy to obtain data on presumed malaria cases at the household level, and 2) data presented is based on participants’ self-reports, which may be associated with socially desirable bias.

Our findings highlight that there is a need to reignite awareness promotion regarding malaria prevention, address misconceptions about malaria symptoms and early signs of pregnancy, and support interventions that promote formal and timely seeking of healthcare. Finally, response to COVID-19 ought to be integrated with malaria efforts by recommitting and integrating COVID-19 measures in the normative living and restrict future barriers to healthcare access.

## Conclusion

The majority of the participants recognized the symptom of malaria, its transmission, and prevention measures. Their malaria prevention experience was highly influenced by the perceived causes and severe adverse effects of malaria infection. Furthermore, the use of home-remedies and non-formal approaches was a common practice. There were reported major negative effects of malaria prevention uptake due to the COVID-19 pandemic.

## Supplementary Information

**Additional file 1.**

## Data Availability

All relevant data are within the paper. The interview guides are included as supplementary materials.

## Abbreviations

FGD: Focus Group Discussion
IDI: In-depth Interview
ITN: Insecticide-Treated mosquito Net
KII: Key Informant Interview
WHO: World Health Organization
