# Supplementary material for: Treatment-seeking and uptake of malaria prevention strategies among pregnant women and caregivers of children under-five years during COVID-19 pandemic in rural communities in South West Uganda: a qualitative study
Source: BMC Public Health. 2022 Feb 21;22:373. doi: 10.1186/s12889-022-12771-3 (PMC8860364; doi:10.1186/s12889-022-12771-3)
Supplement: Supplementary file 1 — Additional file 1. [file 12889_2022_12771_MOESM1_ESM.docx]

## Supplementary material

## File 1: Focus Group Discussion Guide

**FGD ID:** ………………………….. **Parish** ……………………………………………

**Section I: Socio-demographic Characteristics**

1. Age of respondent (__ __) Years

2. Marital status: i. Single ii. Married iii. Separated iv. Divorced v. Widowed vi. Cohabitation

3. Education level: i. Primary ii. Secondary level iii. Tertiary iv. No formal education

4. Religion (optional): i. Christian ii. Muslim iii. Traditional iv. Other (specify)…..............

5. Employment status: i. Employed ii. Self-employed iii. Unemployed

6. Occupation: i. Farmer ii. Civil / Public servant iii. Trader/ businesswoman

7. Number of biological children: ……………………………………….

**Section II: Basic knowledge of malaria**

8. What are the symptoms of malaria? (Where did you learn about it? Prompts: from educational materials, church, family members, VHTs, or friends? Probe for causes, how one locally recognizes that it is malaria, and what he/she did in response to this).

9. For your pregnancy, have you had malaria or since you gave birth, has you or your baby suffered from malaria (Probe for personal experiences from each member in the group)

10. How does someone get an infection with malaria? How is it transmitted?

**Section III: Prevention and treatment against malaria**

11. Do you think malaria is preventable? If yes, how is malaria preventable? (Probe for ways of preventing malaria, where did one get to know the information from, which one is used by a member of the group, how often is this method used, and why it is not used by others. This to be considered for each member of the group).

12. Do you believe that the method you use for malaria prevention is effective? Share with us your own, or your friend's experience?

13. What do you do when you or your baby has suffered from malaria? (Probe for immediate actions before leaving home, where they visited first for emergency care, how long it took them, the condition of the child while at the first facility, and if referred after how long was this).

14. What would you say about the attitude of other village members towards the prevention of malaria using the mentioned method? (Probe for local people, VHTs, healthcare providers).

15. What do you think are the issues with malaria prevention methods that would stop community members from using them? (Probe for as many reasons as possible)

16. What suggestions do you have to improve the use of malaria prevention methods in your village? (Probe for as many suggestions as possible)

##

## File 2: Key Informant Interview Guide (English)

**KII ID:** ……………………………………………………………………………………………..

1. How regularly do pregnant women seek malaria treatment, or bring their babies to treatment in this community? (Probe for frequency and severity)
2. At ANC visits, is malaria emphasized as a major concern for pregnant women?
3. What malaria prevention methods are you aware of in this community? (Probe reports for pregnant women and women with children under-five)
4. What would you say about the attitude of pregnant women towards malaria prevention methods in this community? (Probe for as many views as possible)

Are there any strengths or limitations to malaria prevention methods in this community?

1. What are the important factors that influence the uptake of malaria prevention methods for pregnant women, or those with children under-five? (Probe for as many suggestions as possible)
2. What do you suggest to improve malaria prevention uptake among pregnant women or those with children under-five in this community?
